# STOML2 Alleviates Osteoarthritis by Regulating Mitochondrial Energy Metabolism and Oxidative Stress

**DOI:** 10.1155/genr/9303825

**Published:** 2026-04-17

**Authors:** Fei Xie, Kun Miao, Hao Wu, Xuejun Wu

**Affiliations:** ^1^ Department of Orthopaedics, Fuzhou Second General Hospital, Fuzhou, 350007, China

**Keywords:** mitochondrial dysfunction, osteoarthritis, oxidative stress, STOML2

## Abstract

Osteoarthritis (OA) is associated with chondrocyte dysfunction and cartilage degeneration, but how mitochondrial homeostasis is maintained during OA progression remains incompletely understood. Here, we show that Stomatin‐like protein 2 (STOML2) is downregulated in IL‐1β‐stimulated chondrocytes and in human OA cartilage. STOML2 depletion impairs oxidative phosphorylation, elevates mitochondrial reactive oxygen species, and promotes chondrocyte senescence and ferroptotic cell death. Mechanistically, STOML2 interacts with the mitochondrial Na^+^/Ca^2+^ exchanger (NCLX) to support mitochondrial Ca^2+^ efflux and metabolic stability. Restoring STOML2 enhances NCLX‐dependent Ca^2+^ handling, mitigates mitochondrial Ca^2+^ overload and the glycolytic shift, and improves ATP production. In a rat OA model, intra‐articular delivery of STOML2 attenuates cartilage degeneration and reduces inflammatory changes in the joint. Together, these findings identify a STOML2‐NCLX axis that safeguards mitochondrial Ca^2+^ homeostasis and chondrocyte viability, suggesting STOML2 as a potential therapeutic target for OA.

## 1. Introduction

Osteoarthritis (OA) is a highly prevalent and disabling joint disorder, affecting more than 300 million people worldwide, with a substantial impact on pain, function, and healthcare utilization, particularly in aging populations [1–3]. Clinically, OA most frequently involves the knee, hip, and hand, leading to impaired mobility, reduced work capacity, and considerable healthcare use, and it remains among the leading indications for joint replacement surgery. The population burden increases steeply with age and shows consistent sex differences, with higher prevalence and symptom severity often reported in women, especially in later life [4, 5]. Rather than a purely “wear‐and‐tear” condition, OA is now recognized as a whole‐joint disease involving cartilage, subchondral bone, synovium, and periarticular tissues. Its pathological hallmarks include progressive articular cartilage loss, subchondral bone remodeling, osteophyte formation, and variable degrees of synovitis [6]. Mechanistically, biomechanical stress and inflammatory cues jointly perturb chondrocyte homeostasis, driving extracellular matrix (ECM) catabolism and structural deterioration through upregulation of proteolytic enzymes (e.g., MMPs and ADAMTS) and proinflammatory mediators [7]. Despite the burden of OA, there are currently no approved disease‐modifying OA drugs (DMOADs), and management remains largely symptomatic, focused on pain relief and functional improvement rather than modification of structural disease [8, 9].

Articular cartilage is an avascular and aneural tissue, and its maintenance relies exclusively on the activity and metabolic stability of resident chondrocytes. Under physiological conditions, chondrocytes maintain a balance between anabolic and catabolic processes, preserving ECM integrity. However, under pathological stimuli, such as mechanical overload or inflammatory cytokines such as Interleukin‐1 beta (IL‐1β), this balance is disrupted, leading to chondrocyte dysfunction, matrix degradation, and cartilage destruction [10, 11]. A growing body of evidence implicates mitochondrial dysfunction, oxidative stress, and altered energy metabolism as central features in the pathogenesis of OA [12–14]. Specifically, the shift from oxidative phosphorylation (OXPHOS) to glycolysis and the overproduction of reactive oxygen species (ROS) contribute to chondrocyte senescence, apoptosis, and ferroptosis [12, 15–17].

Stomatin‐like protein 2 (STOML2) is a mitochondrial inner membrane protein involved in the regulation of mitochondrial structure, respiratory chain assembly, and energy metabolism [18–20]. It has been shown to support mitochondrial function in diverse cellular contexts, including stem cells and tumor cells, by promoting OXPHOS and limiting oxidative damage [21]. However, the role of STOML2 in articular cartilage and OA has not yet been investigated. Considering the central role of mitochondria in chondrocyte viability and OA pathology, we hypothesized that STOML2 may function as a key modulator of mitochondrial homeostasis in joint tissues.

Emerging studies have also highlighted the importance of mitochondrial calcium (Ca^2+^) regulation in cellular metabolism and survival. The mitochondrial Na^+^/Ca^2+^ exchanger (NCLX) is the primary efflux channel responsible for maintaining mitochondrial calcium homeostasis, and its dysfunction has been linked to increased ROS production, impaired ATP synthesis, and cell death [22, 23]. Yet, the regulatory mechanisms controlling NCLX expression and activity in chondrocytes remain unclear. Whether STOML2 interacts with NCLX to coordinate calcium flux and metabolic activity has not been explored.

In this study, we first examined the expression of STOML2 in chondrocytes under inflammatory stress and in OA cartilage and found that its levels were markedly reduced. Using in vitro and in vivo models, we investigated the consequences of STOML2 depletion on mitochondrial function, ROS generation, senescence, and ferroptosis. Furthermore, we demonstrated that STOML2 interacts with NCLX to regulate intracellular calcium levels and maintain mitochondrial energy metabolism. Finally, we evaluated the therapeutic potential of STOML2 overexpression in a rat OA model, where it alleviated cartilage degradation and systemic inflammation. These findings uncover a novel role for STOML2 in preserving chondrocyte function and suggest that targeting the STOML2‐NCLX axis may offer a promising strategy for OA treatment.

## 2. Materials and Methods

### 2.1. Primary Chondrocyte Isolation and Culture

Articular chondrocytes were isolated from the knee cartilage of neonatal Sprague−Dawley rats under aseptic conditions. Cartilage tissues were minced and digested with 0.25% trypsin for 30 min, followed by 0.2% Collagenase type II digestion for 4–6 h at 37°C. Isolated chondrocytes were cultured in DMEM/F12 medium supplemented with 10% fetal bovine serum (FBS) and 1% penicillin−streptomycin in a humidified incubator at 37°C with 5% CO_2_.

### 2.2. IL‐1β Treatment

Primary chondrocytes were treated with 10 ng/Ml IL‐1β (PeproTech, Cat# 200‐01 B) for 24 h to induce a degenerative phenotype. Control cells were cultured under identical conditions without IL‐1β.

### 2.3. STOML2 Overexpression and Knockdown

STOML2 overexpression plasmid was transfected into chondrocytes using Lipofectamine 3000 (Thermo Fisher Scientific, Cat# L3000008) following the manufacturer’s instructions. STOML2 knockdown was performed using STOML2‐specific siRNA (Dharmacon, Cat# [30968, 298203]), transfected with Lipofectamine 3000. Control groups received an empty vector or scrambled siRNA. Efficiency was confirmed by quantitative PCR (qPCR) and Western blot.

### 2.4. qPCR

Total RNA was extracted from cells using TRIzol reagent (Invitrogen, Cat# 15596018) according to the manufacturer’s protocol. RNA was reverse‐transcribed into cDNA using the High‐Capacity cDNA Reverse Transcription Kit (Applied Biosystems, Cat# 4368814). qPCR was performed on a QuantStudio 6 Flex Real‐Time PCR System (Applied Biosystems) using SYBR Green Master Mix (Applied Biosystems, Cat# 4309155). The cycling conditions were as follows: Initial denaturation at 95°C for 10 min, followed by 40 cycles of 95°C for 15 s and 60°C for 1 min. Relative gene expression levels were calculated using the 2^–ΔΔCt^ method, with GAPDH serving as the internal reference gene. The primer sequences used were as follows: NCLX forward: 5′‐GCT GAA GAG GAG GAC TGG AA‐3′, reverse: 5′‐CTC AGG AAG TGG GAG AGG TG‐3′; STOML2 forward: 5′‐TGC TGA GGA GGA GAA GAG GA‐3′, reverse: 5′‐GGT CTT GAG GTC CTT GGT TG‐3′; GAPDH forward: 5′‐TGA AGC AGG CAT CTG AGG G‐3′, reverse: 5′‐CGA AGG TGG AAG AGT GGG AG‐3′.

### 2.5. Western Blot Analysis

Proteins were extracted using RIPA buffer (Beyotime, Cat# P0013 B) supplemented with protease and phosphatase inhibitors (Roche, Cat# 04693159001). Equal amounts of protein were separated by SDS−PAGE and transferred onto PVDF membranes (Millipore, Cat# IPVH00010). Membranes were blocked with 5% nonfat milk and incubated overnight at 4°C with the following primary antibodies diluted in blocking buffer: STOML2 (Abcam, Cat# ab230973, 1:1000), NCLX (Proteintech, Cat# 25764‐1‐AP, 1:1000), Matrix metalloproteinase‐9 (MMP9) (Abcam, Cat# ab38898, 1:1000), p16^INK4a^ (Abcam, Cat# ab108349, 1:1000), p21 (Cell Signaling Technology, Cat# 2947, 1:1000), p53 (Abcam, Cat# ab32389, 1:1000), GPX4 (Abcam, Cat# ab125066, 1:1000), FTH1 (Abcam, Cat# ab65080, 1:1000), SLC7A11 (Abcam, Cat# ab175186, 1:1000), and β‐actin (Cell Signaling Technology, Cat# 4970, 1:5000). After washing, membranes were incubated with HRP‐conjugated secondary antibodies (anti‐rabbit IgG, Cell Signaling Technology, Cat# 7074, 1:5000; anti‐mouse IgG, Cell Signaling Technology, Cat# 7076, 1:5000) for 1 h at room temperature. Protein bands were visualized using enhanced chemiluminescence reagents (Thermo Fisher, Cat# 34577).

### 2.6. Immunofluorescence (IF) Staining

Cells were fixed in 4% paraformaldehyde, permeabilized with 0.1% Triton X‐100, and blocked in 5% BSA. Primary antibodies against STOML2 were applied overnight, followed by fluorophore‐conjugated secondary antibodies. Nuclei were counterstained with DAPI and imaged under a fluorescence microscope.

### 2.7. ROS Assay

ROS levels were detected with the DCFH‐DA fluorescent probe (Sigma‐Aldrich, Cat# D6883). Cells were incubated with 10 μM DCFH‐DA for 30 min at 37°C, washed, and then analyzed by fluorescence microscopy and flow cytometry (BD FACSCanto II).

### 2.8. Intracellular Calcium Measurement

Intracellular calcium concentration was quantitatively measured using the Calcium Assay Kit (Abcam, Cat# ab102505) according to the manufacturer’s instructions. Cells were loaded with the calcium‐sensitive fluorescent probe and incubated at 37°C. Fluorescence intensity was detected using a microplate reader (excitation/emission: 485/535 nm), and absolute calcium concentrations were calculated based on a standard calibration curve provided by the kit.

### 2.9. Lactate and ATP Measurements

Extracellular lactate was quantified using a Lactate Assay Kit (BioVision, Cat# K607‐100). Intracellular ATP content was measured using an ATP Assay Kit (Promega, Cat# G7571) based on luciferase bioluminescence.

### 2.10. Co‐Immunoprecipitation (Co‐IP)

Protein lysates were incubated with anti‐STOML2 (Abcam, Cat# ab230973) or anti‐NCLX (Proteintech, Cat# 25764‐1‐AP) antibodies overnight at 4°C, followed by incubation with Protein A/G agarose beads (Thermo Fisher, Cat# 20421) for 2 h. Immunoprecipitates were washed, eluted, and analyzed by Western blot.

### 2.11. Rat OA Model and Intra‐Articular Injection

OA was induced in 8‐week‐old male Sprague–Dawley rats using anterior cruciate ligament transection (ACLT). Animals were allocated into three groups: Sham (control, *n* = 3), ACLT + control lentivirus (*n* = 3), and ACLT + STOML2‐overexpressing lentivirus (*n* = 3). Group size was determined based on our previous experience with the ACLT model and feasibility considerations. Randomization was performed after surgery using a computer‐generated random number list (simple randomization), and group assignments were concealed until the time of intra‐articular injection. Intra‐articular injections of STOML2‐overexpressing lentivirus or the corresponding control vector were administered according to the assigned group. At 8 weeks postsurgery, knee joints were harvested for histological and molecular analyses.

### 2.12. Histological Analysis and OARSI Scoring

Knee joints were fixed, decalcified, paraffin‐embedded, sectioned, and stained with hematoxylin and eosin (H&E). OA severity was assessed using the OA Research Society International (OARSI) scoring system. Histological scoring was performed in a blinded manner by two independent observers who were unaware of the group allocation. For each joint, multiple serial sections spanning the weight‐bearing region were evaluated, and the final OARSI score was calculated as the mean of the observers’ scores. In cases of discrepancy, scores were re‐evaluated jointly to reach consensus.

## 3. ELISA

Serum cytokine levels of IL‐6, IL‐1β, and TNF‐α were measured using ELISA kits from R&D Systems: IL‐6 (Cat# DY406), IL‐1β (Cat# DY201), and TNF‐α (Cat# DY210), following the manufacturers’ protocols.

### 3.1. Statistical Analysis

All experiments were conducted in triplicate. Data are presented as mean ± standard deviation (SD). Statistical analyses were performed using GraphPad Prism 9.0. One‐way ANOVA followed by Tukey’s post hoc test was used to compare multiple groups. *p* < 0.05 was considered statistically significant.

## 4. Results

### 4.1. STOML2 Expression is Downregulated in IL‐1β‐Induced Degenerative Chondrocytes

Primary chondrocytes were isolated and subsequently stimulated with IL‐1β to induce a degenerative phenotype. qPCR and Western blot analyses were performed to assess the expression levels of STOML2. As shown in Figures 1(a) and 1(b), both mRNA and protein levels of STOML2 were significantly downregulated in IL‐1β‐treated degenerative chondrocytes compared to controls. IF staining further confirmed the reduced expression of STOML2 in degenerative chondrocytes (Figure 1(c)). These findings collectively indicate that STOML2 expression is markedly decreased in chondrocytes under inflammatory and degenerative conditions.

FIGURE 1STOML2 expression is downregulated in IL‐1β‐induced degenerative chondrocytes. (a) qPCR analysis of STOML2 mRNA expression in primary chondrocytes following IL‐1β treatment compared to control cells. (b) Western blot analysis of STOML2 protein expression in primary chondrocytes following IL‐1β treatment compared to control cells. (c) Immunofluorescence staining of STOML2 in control and IL‐1β‐treated chondrocytes. Scale bar = 25 μm.

**Figure:**
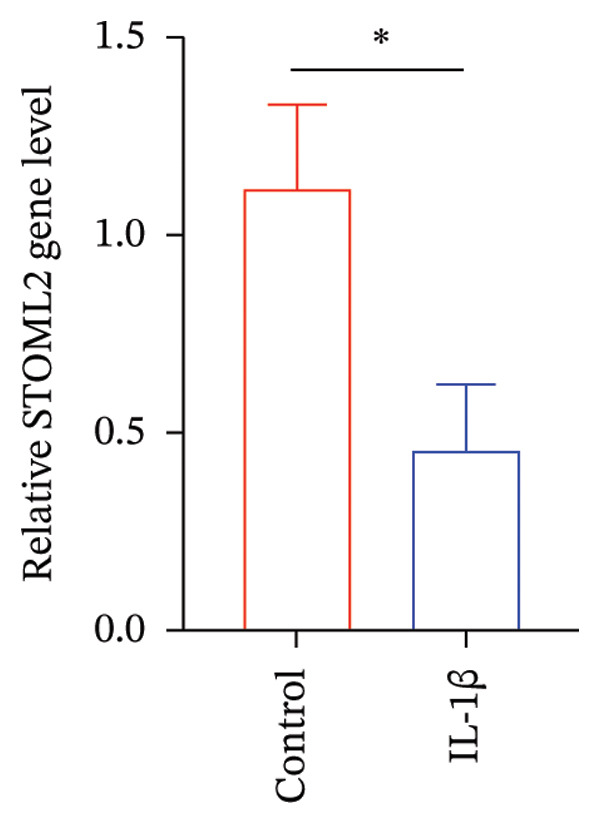
(a)

**Figure:**
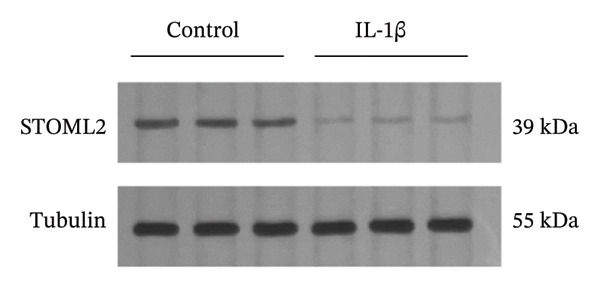
(b)

**Figure:**
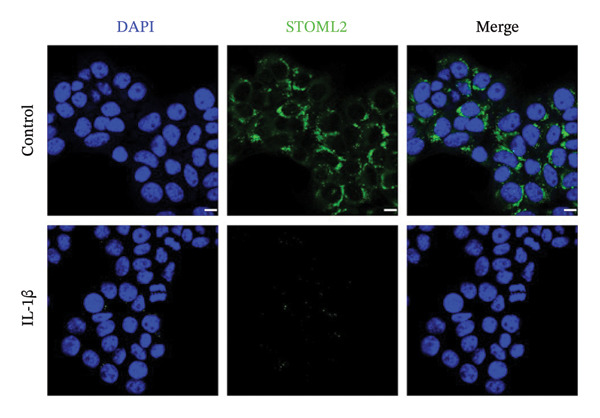
(c)

### 4.2. STOML2 Knockdown Disrupts Mitochondrial Function, Increases Oxidative Stress, and Promotes Senescence and Ferroptosis in Chondrocytes

To further investigate the functional role of STOML2 in chondrocyte physiology, we examined the effects of STOML2 knockdown on oxidative stress and mitochondrial energy metabolism. Knockdown of STOML2 in chondrocytes significantly impaired ATP production, as evidenced by a marked reduction in mitochondrial function parameters (Figure 2(a)). To assess the impact of STOML2 depletion on cellular senescence, Western blot analysis was performed to measure the expression levels of senescence‐associated markers including p16^INK4a^, p21, and p53. Results demonstrated that STOML2‐deficient chondrocytes exhibited increased expression of all three markers, indicating a pronounced senescent phenotype (Figure 2(b)). ROS generation was subsequently evaluated using the DCFH‐DA fluorescent probe. A significant increase in ROS levels was detected following STOML2 knockdown (Figures 2(c) and 2(d)), and this finding was further validated by flow cytometry, corroborating the elevated oxidative stress state in these cells. In addition, we assessed the expression of ferroptosis‐associated proteins, including GPX4, FTH1, and SLC7A11. Western blot analysis revealed that STOML2 knockdown resulted in decreased expression of all three markers, suggesting that loss of STOML2 may predispose chondrocytes to ferroptosis. Taken together, these findings demonstrate that STOML2 plays a critical role in maintaining mitochondrial function, redox homeostasis, and cell viability in chondrocytes, and its depletion leads to metabolic dysregulation, enhanced oxidative stress, cellular senescence, and ferroptosis.

FIGURE 2STOML2 knockdown disrupts mitochondrial function, increases oxidative stress, and promotes senescence and ferroptosis in chondrocytes. (a) Measurement of ATP production in chondrocytes after STOML2 knockdown using metabolic assays. (b) Western blot detection of senescence‐associated proteins p16INK4a, p21, and p53 in STOML2‐deficient chondrocytes. (c and d) Intracellular ROS levels detected using the DCFH‐DA fluorescent probe, with corresponding fluorescence quantification. Scale bar = 100 μm. (e) Flow cytometric analysis of ROS levels in chondrocytes following STOML2 knockdown. (f) Western blot analysis of ferroptosis‐related proteins GPX4, FTH1, and SLC7A11 in chondrocytes after STOML2 knockdown.

**Figure:**
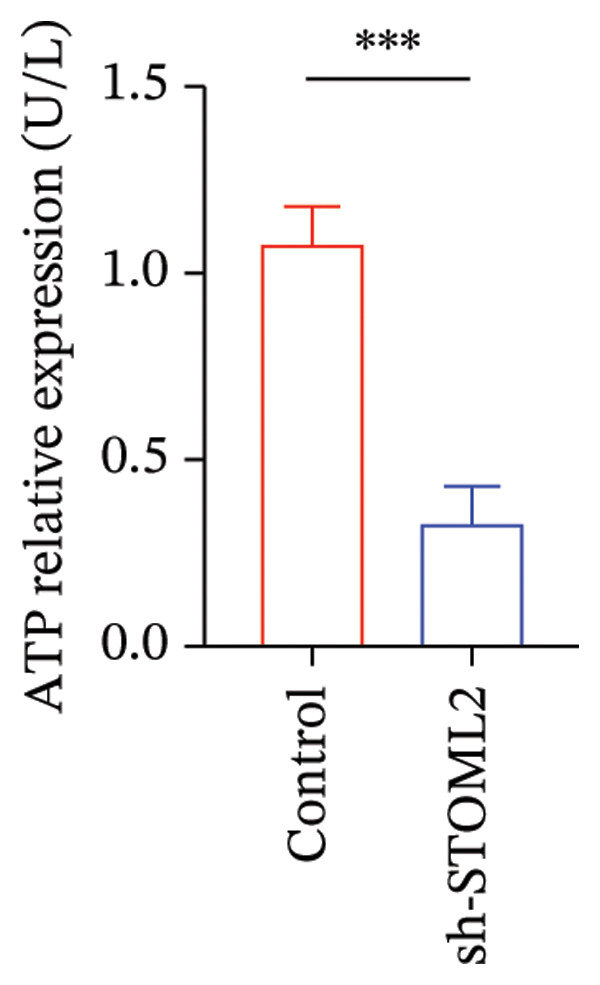
(a)

**Figure:**
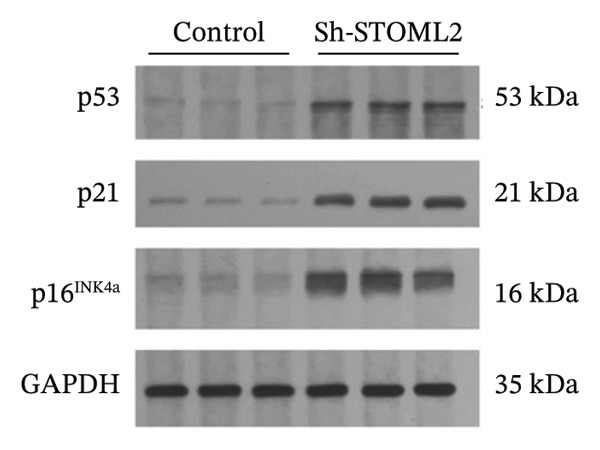
(b)

**Figure:**
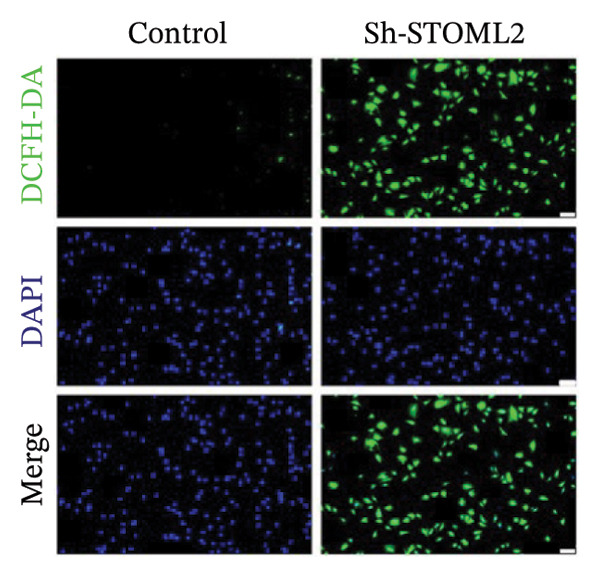
(c)

**Figure:**
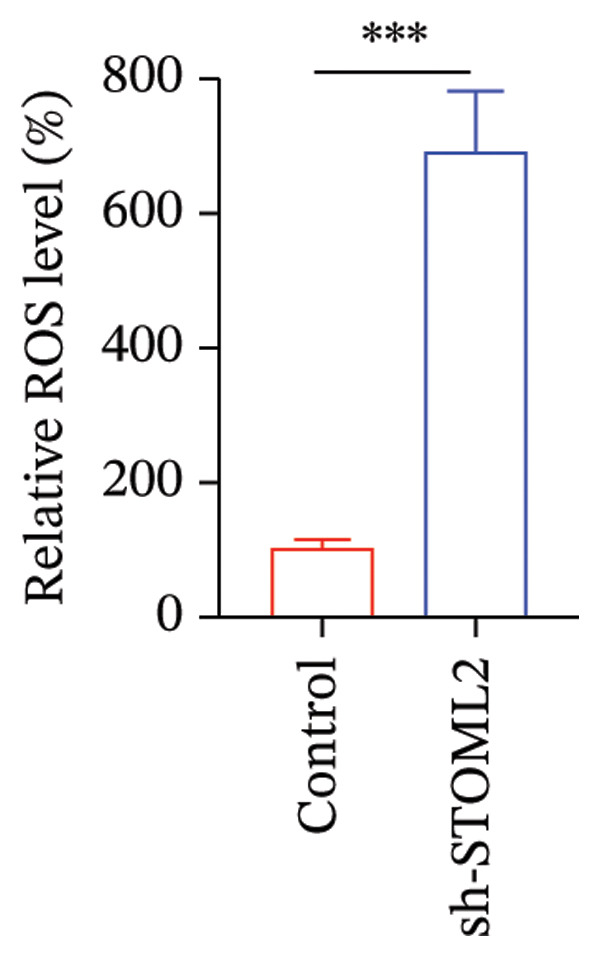
(d)

**Figure:**
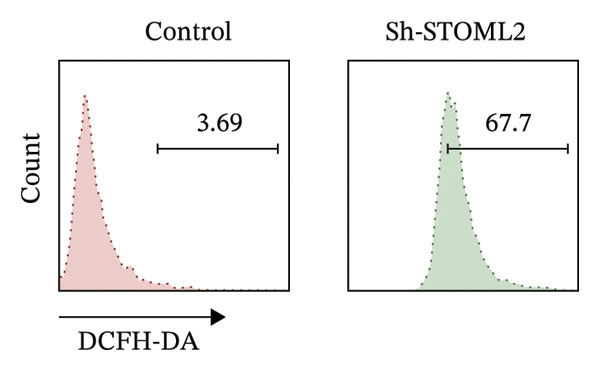
(e)

**Figure:**
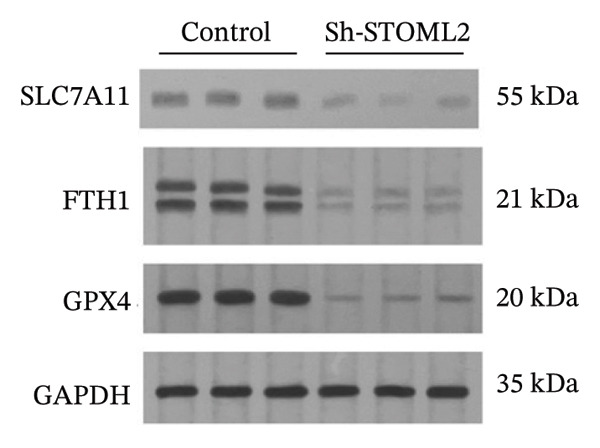
(f)

### 4.3. STOML2 Overexpression Attenuates Cartilage Degeneration and Inflammation in a Rat OA Model

To further elucidate the in vivo relevance of STOML2 in OA, we established a rat OA model to evaluate whether overexpression of STOML2 could attenuate disease progression. Initially, we confirmed that STOML2 expression was significantly reduced in OA cartilage tissues, as determined by both qPCR and Western blot analyses (Figures 3(a) and 3(b)). Upon intra‐articular administration of a STOML2‐overexpressing vector, the expression of STOML2 was markedly upregulated, confirming successful gene delivery. Histological analysis using H&E staining, along with OARSI scoring, demonstrated that STOML2 overexpression substantially alleviated cartilage degradation and reduced OA severity, suggesting a protective effect against disease progression (Figures 3(c) and 3(d)). Western blot analysis further revealed that MMP9, a key catabolic enzyme involved in cartilage breakdown, was significantly upregulated in OA cartilage. Notably, STOML2 overexpression led to a marked reduction in MMP9 expression levels (Figure 3(e)), indicating suppression of cartilage matrix degradation. In addition, ELISA assays showed that systemic levels of proinflammatory cytokines, including IL‐6, IL‐1β, and TNF‐α, were significantly elevated in OA rats. STOML2 overexpression effectively decreased the serum concentrations of these inflammatory mediators (Figure 3(f)), further supporting its anti‐inflammatory role in vivo. Collectively, these in vivo results suggest that STOML2 overexpression can mitigate OA progression by reducing cartilage degradation and systemic inflammation, highlighting its potential as a therapeutic target for OA.

FIGURE 3STOML2 overexpression attenuates cartilage degeneration and inflammation in a rat osteoarthritis model. (a and b) qPCR and Western blot analyses of STOML2 expression in cartilage samples from normal, OA, and STOML2‐overexpression groups. (c) Histological staining of knee joint cartilage using hematoxylin and eosin (H&E) in different experimental groups. Scale bar = 50 μm. (d) OARSI scoring of cartilage sections based on histological evaluation. (e) Western blot analysis of MMP9 protein levels in cartilage tissues across treatment groups. (f) ELISA quantification of serum levels of IL‐6, IL‐1β, and TNF‐α in rats subjected to different treatments.

**Figure:**
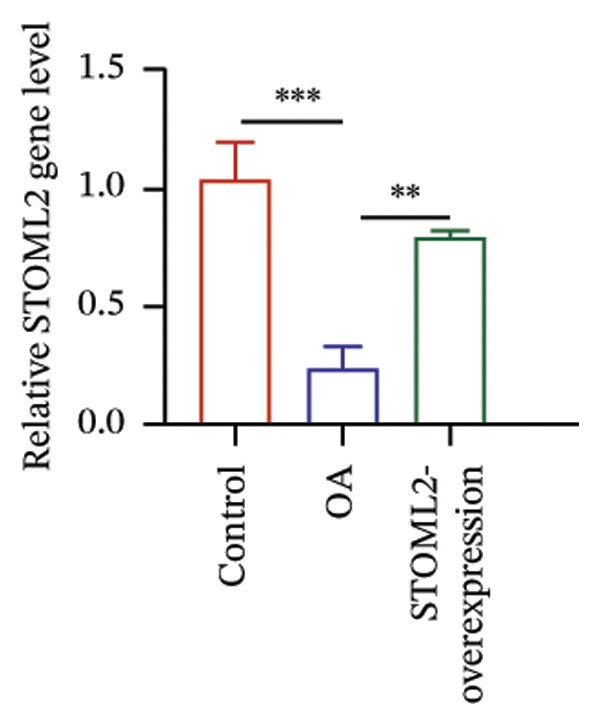
(a)

**Figure:**
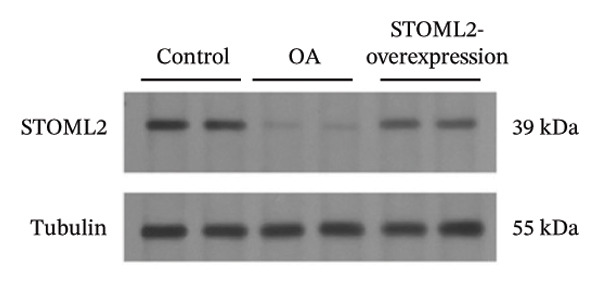
(b)

**Figure:**
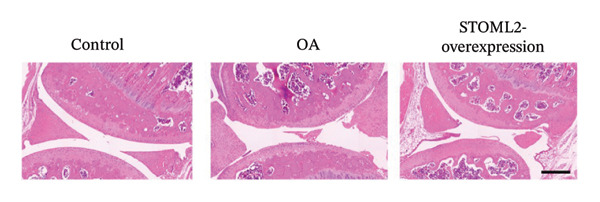
(c)

**Figure:**
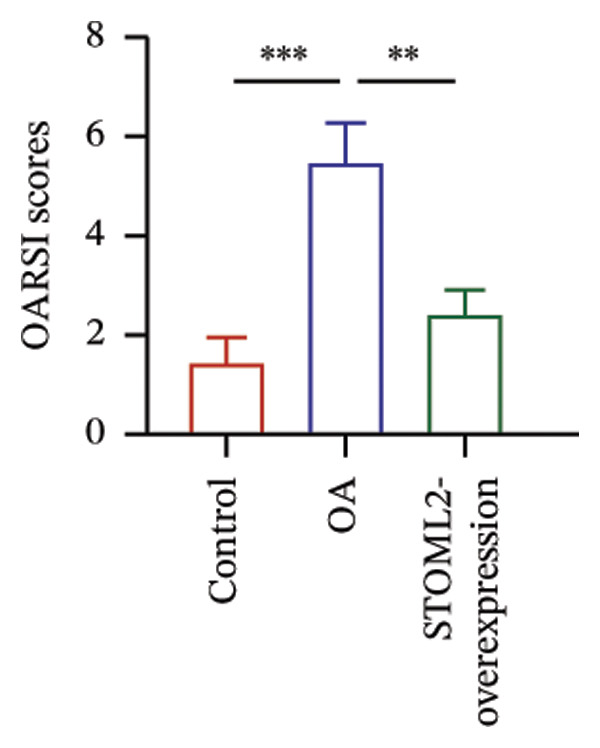
(d)

**Figure:**
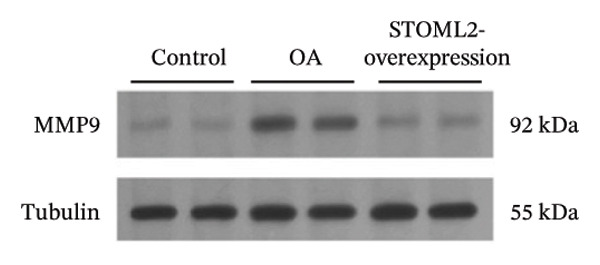
(e)

**Figure:**
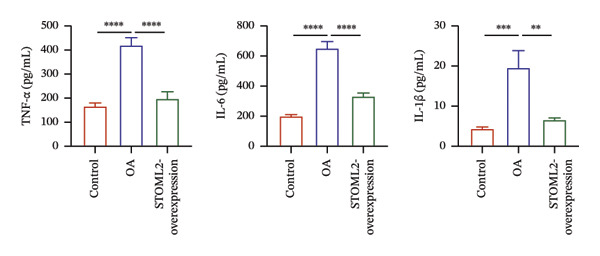
(f)

## 5. STOML2 Regulates Mitochondrial Energy Metabolism by Interacting With NCLX to Modulate Calcium Homeostasis

To investigate how STOML2 regulates mitochondrial Ca^2+^ homeostasis and bioenergetics under inflammatory stress, we first examined the relationship between STOML2 and the mitochondrial Na^+^/Ca^2+^ exchanger NCLX. Co‐immunoprecipitation assays demonstrated a specific association between STOML2 and NCLX, as evidenced by reciprocal pull‐down of STOML2 with anti‐NCLX and of NCLX with anti‐STOML2 (Figure 4(a)), supporting that STOML2 engages NCLX in chondrocytes. We then asked whether STOML2 affects NCLX abundance. IL‐1β stimulation markedly reduced NCLX expression at both the mRNA and protein levels, whereas STOML2 overexpression restored NCLX expression (Figures 4(b) and 4(c)), indicating that STOML2 positively regulates NCLX expression/maintenance in the inflammatory setting. Consistent with this, IL‐1β induced a pronounced increase in cytosolic Ca^2+^, which was significantly attenuated by STOML2 overexpression (Figure 4(d)), suggesting improved Ca^2+^ handling when STOML2 is restored. Given the close coupling between Ca^2+^ dysregulation and metabolic reprogramming, we further assessed lactate production and ATP content. IL‐1β increased lactate accumulation and decreased ATP levels, whereas STOML2 overexpression reversed these changes (Figures 4(e) and 4(f)), consistent with mitigation of the glycolytic shift and preservation of mitochondrial bioenergetics. Together, these data suggest that STOML2, through its association with NCLX and preservation of NCLX expression under IL‐1β stress, supports Ca^2+^ homeostasis and maintains mitochondrial energy metabolism in chondrocytes.

FIGURE 4STOML2 regulates mitochondrial energy metabolism by interacting with NCLX to modulate calcium homeostasis. (a) Co‐immunoprecipitation (Co‐IP) analysis of the interaction between STOML2 and the mitochondrial sodium−calcium exchanger NCLX. (b) qPCR analysis of NCLX mRNA expression in chondrocytes under the indicated conditions. (c) Western blot analysis of NCLX protein levels across different treatment groups. (d) Measurement of intracellular calcium concentration in chondrocytes under the indicated conditions. (e) Measurement of lactate production to evaluate glycolytic activity in chondrocytes under different experimental conditions. (f) Quantification of ATP levels in chondrocytes under different experimental conditions.

**Figure:**
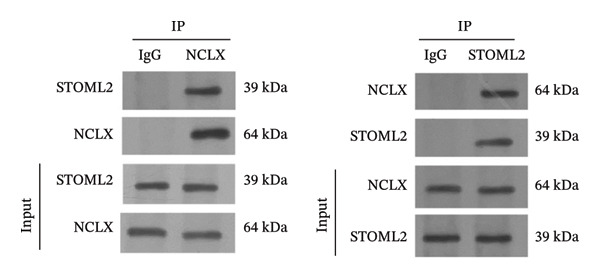
(a)

**Figure:**
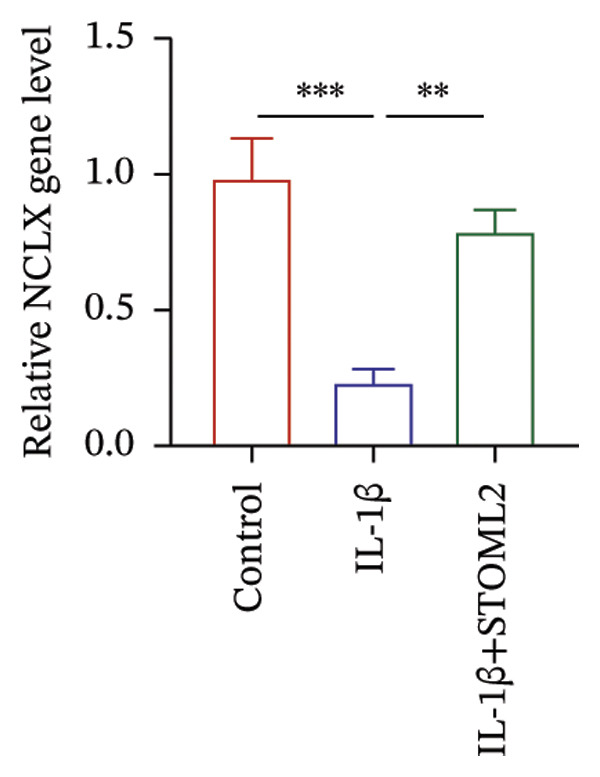
(b)

**Figure:**
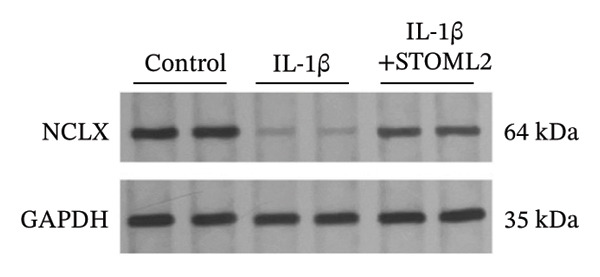
(c)

**Figure:**
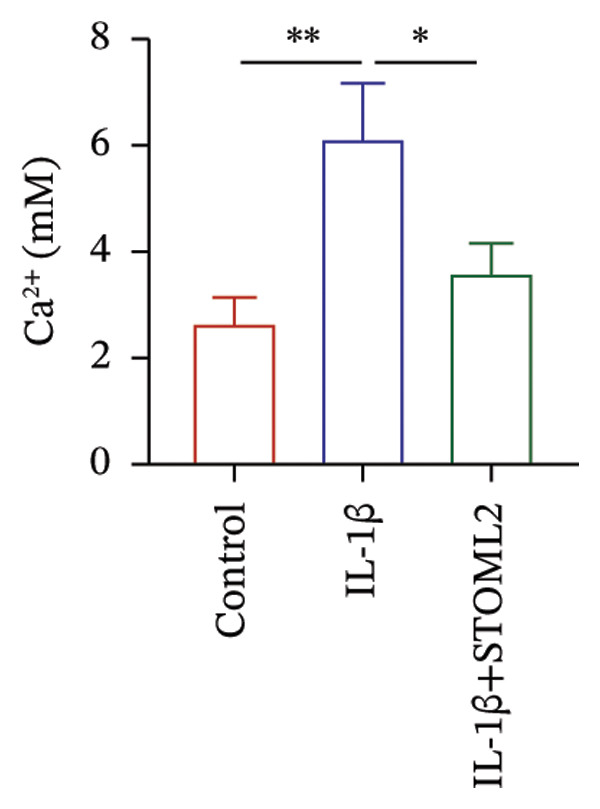
(d)

**Figure:**
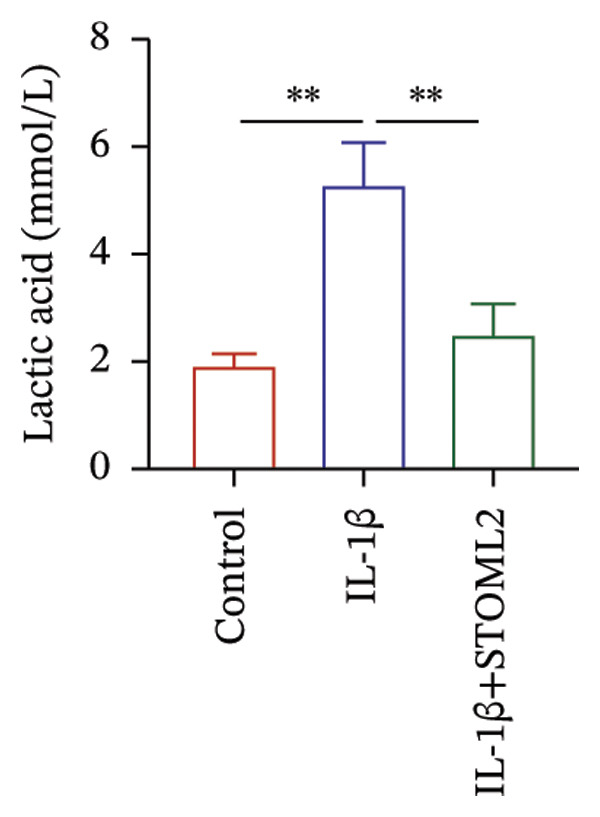
(e)

**Figure:**
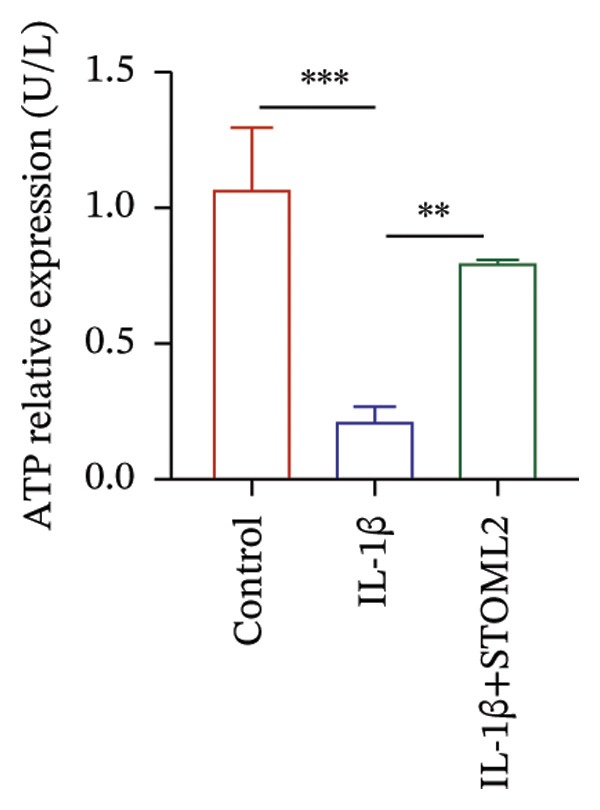
(f)

## 6. Discussion

In this study, we identified STOML2 as a critical regulator of mitochondrial energy metabolism and chondrocyte viability under inflammatory and degenerative conditions. STOML2 expression was significantly downregulated in IL‐1β−treated chondrocytes and OA cartilage, implicating its potential involvement in OA pathogenesis. Functionally, silencing of STOML2 impaired mitochondrial OXPHOS, as evidenced by reduced ATP production, and induced a compensatory metabolic shift toward glycolysis, reflected by increased lactate accumulation. In parallel, STOML2 deficiency led to elevated ROS generation, enhanced expression of senescence markers (p16^INK4a^, p21, and p53), and downregulation of key ferroptosis‐related proteins (GPX4, FTH1, and SLC7A11), collectively promoting oxidative stress, cellular senescence, and susceptibility to ferroptosis. These findings underscore the multifaceted role of STOML2 in preserving mitochondrial function, metabolic balance, and redox homeostasis in chondrocytes.

In vivo, overexpression of STOML2 in a rat model of OA significantly alleviated cartilage degradation, as demonstrated by histological analysis and reduced OARSI scores. This chondroprotective effect was accompanied by suppression of MMP9 and a marked decrease in systemic inflammatory cytokines, including IL‐1β, IL‐6, and TNF‐α. Mechanistically, STOML2 was found to physically interact with the mitochondrial sodium−calcium exchanger NCLX. This interaction was disrupted under IL‐1β stimulation but restored upon STOML2 overexpression. Restoration of the STOML2‐NCLX interaction normalized intracellular calcium levels, which is essential for sustaining mitochondrial membrane potential, calcium‐dependent enzymatic activity, and ATP synthesis through OXPHOS.

Taken together, our findings establish STOML2 as a central metabolic regulator that safeguards mitochondrial OXPHOS, limits aberrant glycolysis, and maintains intracellular calcium and redox balance in chondrocytes under inflammatory stress. Loss of STOML2 drives metabolic reprogramming and oxidative damage, contributing to the pathophysiology of OA. Moreover, therapeutic STOML2 overexpression in vivo mitigates cartilage degradation and inflammation, supporting its translational potential in OA treatment. Notably, while mitochondrial dysfunction and oxidative stress have been implicated in OA, upstream regulators that mechanistically couple NCLX‐associated Ca^2+^ homeostasis to chondrocyte bioenergetics and cell fate remain incompletely defined. Our study, therefore, highlights a previously unrecognized STOML2‐NCLX–calcium axis linking mitochondrial calcium homeostasis to energy metabolism and cartilage integrity and suggests that targeting this pathway may represent a promising strategy for the development of mitochondrial‐targeted therapies for OA.

## 7. Limitations

This study has several limitations. First, although STOML2 was consistently downregulated in IL‐1β‐stimulated chondrocytes and OA cartilage, the upstream mechanisms responsible for this dysregulation remain undefined. Future work should determine whether inflammatory signaling, mechanical loading–responsive pathways, or epigenetic regulation drive STOML2 suppression during OA progression. Second, while intra‐articular delivery of STOML2‐overexpressing lentivirus ameliorated cartilage degeneration in the rat ACLT model, we did not systematically evaluate long‐term safety, durability of expression, or potential off‐target and immune effects associated with gene delivery; these issues will be important for translational development.

## Author Contributions

Fei Xie and Xuejun Wu designed the study, collated data, carried out data analyses, and produced the initial draft of the manuscript. Kun Miao contributed to collecting specimens and tissue sections.

## Funding

This study was supported by the Natural Science Foundation of Fujian Province (2023J01121466) and the Fujian Provincial Clinical Medical Research Center for First Aid and Rehabilitation in Orthopedic Trauma (2020Y2014).

## Disclosure

All authors read and approved the final manuscript.

## Consent

The authors confirm that written consent for publication has been obtained from all participants involved in this study.

## Conflicts of Interest

The authors declare no conflicts of interest.

## Data Availability

All data of this study are included within the article.
