# Correction to “STOML2 Alleviates Osteoarthritis by Regulating Mitochondrial Energy Metabolism and Oxidative Stress”

**DOI:** 10.1155/genr/9865785

**Published:** 2026-08-12

F. Xie, K. Miao, H. Wu, and X. Wu, “STOML2 Alleviates Osteoarthritis by Regulating Mitochondrial Energy Metabolism and Oxidative Stress,” *Genetics Research* 2026 (2026): 9303825, https://doi.org/10.1155/genr/9303825.

In the article titled “STOML2 Alleviates Osteoarthritis by Regulating Mitochondrial Energy Metabolism and Oxidative Stress,” an error occurred in the Funding section. The grant number for the Natural Science Foundation of Fujian Province was incorrectly published as 2023J01121466. The correct Grant number is 2023J011540.

The corrected Funding section is as follows:

**Funding**

This study was supported by the Natural Science Foundation of Fujian Province (2023J011540) and the Fujian Provincial Clinical Medical Research Center for First Aid and Rehabilitation in Orthopedic Trauma (2020Y2014).

We apologize for this error.

